# Adjuvant Treatments of Adult Melanoma: A Systematic Review and Network Meta-Analysis

**DOI:** 10.3389/fonc.2022.926242

**Published:** 2022-06-17

**Authors:** Mingyi Jing, Yi Cai, Jing Shi, Xufan Zhang, Baohua Zhu, Fan Yuan, Jie Zhang, Min Xiao, Mingling Chen

**Affiliations:** ^1^ Department of Dermatology, Hospital of Chengdu University of Traditional Chinese Medicine, Chengdu, China; ^2^ Chengdu University of Traditional Chinese Medicine, Chengdu, China; ^3^ Department of Oncology, Hospital of Chengdu University of Traditional Chinese Medicine, Chengdu, China; ^4^ Department of Internal Medicine, Hospital of Chengdu University of Traditional Chinese Medicine, Chengdu, China; ^5^ Department of Nuclear Medicine, Hospital of Chengdu University of Traditional Chinese Medicine, Chengdu, China; ^6^ Department of Urology & Andrology, Hospital of Chengdu University of Traditional Chinese Medicine, Chengdu, China

**Keywords:** melanoma, adjuvant treatment, network meta-analysis, nivolumab, ipilimumab, trametinib

## Abstract

Multiple treatments of unresectable advanced or metastatic melanoma have been licensed in the adjuvant setting, causing tremendous interest in developing neoadjuvant strategies for melanoma. Eligible studies included those that compared overall survival/progression-free survival/grade 3 or 4 adverse events in patients with unresectable advanced or metastatic melanoma. Seven eligible randomized trials with nine publications were included in this study. Direct and network meta-analysis consistently indicated that nivolumab+ipilimumab, nivolumab, and trametinib could significantly improve overall survival and progression-free survival compared to ipilimumab in advanced melanoma patients. Compared to ipilimumab, nivolumab, dacarbazine, and ipilimumab+gp100 had a reduced risk of grade 3/4 adverse reactions. The nivolumab+ipilimumab combination had the highest risk of adverse events, followed by ipilimumab+dacarbazine and trametinib. Combination therapy was more beneficial to improve overall survival and progression-free survival than monotherapy in advanced melanoma treatment, albeit at the cost of increased toxicity. Regarding the overall survival/progression-free survival, ipilimumab+gp100 ranked below ipilimumab+dacarbazine and nivolumab+ipilimumab, although it had a smaller rate of grade 3 or 4 AEs than other treatments (except nivolumab). Nivolumab is the optimum adjuvant treatment for unresectable advanced or metastatic melanoma with a good risk-benefit profile. In order to choose the best therapy, clinicians must consider the efficacy, adverse events, and physical status.

## Introduction

Melanoma is a type of skin cancer that arises from melanocytes. Advanced melanoma, including metastatic and unresectable cases, has consistently been one of the most lethal cancers in the world. Patients with metastatic melanoma have a 5-year survival rate of less than 16% (1). Patients with stage IV melanoma have a 6.2-month median overall survival (OS) and a 25.5% 1-year survival rate (2). Over the last several decades, the worldwide incidence of malignant melanoma has risen steadily (3). According to the American Cancer Society’s most recent epidemiological data, the number of new invasive melanoma cases detected each year has grown by 31% over the last decade (2012–2022). Besides, it is predicted that the number of melanoma mortality would rise by 6.5% in 2022 (4). The etiology of melanoma is related to the human body and environment. Ultraviolet radiation, skin phototype, pigmented nevi, pesticide usage, prolonged sun exposure and sunburn, geographical location, heredity, genetic factors, immunosuppressive conditions, and non-melanoma skin cancer are all risk factors for the occurrence of melanoma (3). Given all of that, combined with the recently developed research, the main pathogenesis of melanoma can be considered as excessive ultraviolet exposure, gene mutations (BRAF, NRAS and NF1 gene mutations) and molecular signaling pathways (MAPK pathway and PI3K pathway), etc. (3, 5–7)

Melanoma is cancer that is basically incurable. To date, several treatment options for melanoma have been developed, including surgery, chemotherapy, radiotherapy, hormone therapy, targeted therapy, etc. Cancer cells do not migrate to distant cells and tissues in the early stages of cancer, hence surgery is commonly utilized at this stage. In contrast, surgery is not recommended for advanced cancer due to its invasiveness (8–10). Adjuvant treatments such as chemotherapy, radiotherapy, hormone therapy and targeted therapy are commonly used in the treatment of advanced melanoma. In addition to these conventional therapies, mesoporous bioactive glasses (MBGs, a special class of bioactive glasses) play a role in innovative cancer treatment methodologies. Due to their outstanding stability and high drug loading capacity, MBGs are an excellent candidate for the development of advanced drug delivery systems with sustained and/or controlled drug release profiles in cancer treatment (11). Moreover, nanotechnology is a unique material with transformation potential in cancer diagnosis, screening and treatment. The application of nanotechnology enables drugs and active biomolecules to identify and target tumor cells more accurately and effectively (12). Another review discusses more research and development of tannic acid-incorporated medical applications, with cancer therapy being a particular focus of this article (13). These new advanced materials have bright prospects in the treatment of melanoma.

Nevertheless, immune checkpoint inhibitors and targeted therapies remain the most common adjuvant treatments for unresectable advanced or metastatic melanoma.The Food and Drug Administration (FDA) of the United States has authorized several novel adjuvant treatments for unresectable advanced or metastatic melanoma since 2011. For instance, kinase inhibitors (targeting mutant BRAF or MEK) inhibit driving pathways in around half of the melanoma patients (14) and immune checkpoint inhibitors (targeting CTLA-4, PD-1, or PD-L1) can kill melanoma cells. These adjuvant treatments have significantly altered the therapeutic landscape. The most often used checkpoint inhibitors are monoclonal antibodies that block the CTLA-4 (ipilimumab) and PD-1 (pembrolizumab and nivolumab) pathways (15, 16). The FDA has approved ipilimumab which can increase the overall survival (OS) rate of advanced melanoma patients, in which approximately 11% of patients have objective responses (17, 18). Similarly, pembrolizumab and nivolumab were authorized by the FDA as the first anti-PD-1 (CD279) directed monoclonal antibodies in the treatment of advanced cancers (namely advanced or metastatic melanoma). Nivolumab has been proven to increase progression-free survival (PFS) and OS in unresectable melanoma patients (19). Intriguingly, research has shown that ipilimumab combined with nivolumab had better efficiency with higher response rates and long-term OS rates than monotherapies. Unfortunately, the combination therapy possessed a high toxicity rate, making it ineffective for treating advanced melanoma (20, 21). Pembrolizumab had a 4-year OS rate of 37%, while the 3-year OS rate of ipilimumab combined with nivolumab was 58% (22, 23). It was observed that the median OS was 72.1 months for the combination of ipilimumab and nivolumab, 19.9 months for ipilimumab, and 36.9 months for nivolumab after 6.5 years of follow-up (24). Overall, ipilimumab combined with nivolumab has become a gold standard for treating metastatic melanoma. In addition, targeted therapies, including BRAF and MEK inhibitors (BRAFi/MEKi), are authorized for patients with BRAF V600-mutant melanoma. BRAFi/MEKi is beneficial for significantly prolonging OS, with dabrafenib and trametinib having 44% 3-year OS rates (25).

Despite the breakthrough advances in treating unresectable advanced or metastatic melanoma, the best course of therapy remains unclear. Interestingly, no previous similar article on patients with unresectable advanced or metastatic melanoma was found. The efficacy and side effects of these medications alone, as well as their combination usage, have not been well assessed, and further research is necessary to confirm the efficacy and safety of adjuvant treatments for advanced melanoma. Additionally, it is difficult to obtain a comprehensive and satisfactory synthesis of current scientific evidence on adjuvant treatments by traditional meta-analysis methods, owing to a paucity of head-to-head trials. Network meta-analysis (NMA) is a statistical approach that assesses numerous treatments in a single study by incorporating direct and indirect evidence from randomized controlled trials in a network of randomized controlled trials (RCT) (26, 27). Therefore, we employed an NMA technique for the major adjuvant treatments in terms of OS, PFS, and adverse events (AEs) of grade 3 or 4 and obtained the optimum adjuvant treatment for advanced melanoma.

## Methods

The PRISMA guidelines (Preferred Reporting Items for Systematic Reviews and Meta-Analysis) are the basis for implementing the network meta-analysis. Details of the methodology and reporting follow the PRISMA guidelines. The PROSPERO registration number is CRD42021291959.

### Literature Search and Selection Criteria

We searched PubMed, Embase, and Cochrane Library databases from their inception to January 2021. **Supplementary Table S1** outlines the comprehensive search strategy. The search keywords used were: “Melanoma”, “Chemotherapy, Adjuvant”, “Molecular Targeted Therapy”, “Molecular Targeted Therapies”, “Vemurafenib”, “Ipilimumab”, “Nivolumab”, “pembrolizumab”, “Immune Checkpoint Inhibitors”, “Cytokine-Induced Killer Cells”, “randomized controlled trial (RCT)”, etc.

Two investigators performed the selection of studies independently, and they included studies with no language restrictions to limit publication bias. Then, the duplicate literature, irrelevant literature, and incomplete articles were excluded. Any disagreements were resolved by the third investigator. The inclusion criteria in this study are as follows (1): Adult patients with no prior systemic therapies, unresectable or metastatic histologically confirmed stage III or IV wild-type BRAF melanoma (2); The patient received adjuvant therapy (at least one treatment arm) (3); reported the OS/PFS/AE. The exclusion criteria are as follows (1): the included criteria for melanoma mentioned above are not used (2); case reports, reviews, comments, letters, conference reports, duplicate reports, or unfinished studies (3); there was no full text available, and there were inadequate data in the literature (4); trials without a control arm.

### Data Extraction and Quality Assessment

Two independent investigators read all eligible literature, then they extracted the available data, including the name of the study, name of the first author, publication year, trial phase, treatment arms, number and characteristics of enrolled patients, regimens of adjuvant treatments, the number of patients per treatment arm, follow-up period, oncological results, grade AEs results. Afterward, we retrieved the hazard ratios (HRs) and 95% CIs correlated with OS and PFS, and grade 3/4 AEs rate.

Two researchers utilized the Cochrane risk-of-bias tool to assess the quality of individual studies. Based on seven quality assessment projects, including random sequence generation, allocation concealment, blinding of participants and personnel, blinding of outcome assessment, incomplete outcomes, selective reporting, and other bias, each of the studies was classified as having a low, high, or unclear risk of bias. Any discrepancies were settled by discussions with a third researcher in this process.

### Outcomes

For the primary outcome, we extracted hazard ratios (HR) for OS and PFS) as well as the 95% confidence intervals (CIs). The secondary outcome was adverse events (AEs) in grades 3 or 4.

### Data Analyses

HR for OS, PFS, and 95% CIs were utilized as summary statistics to assess the efficacy of adjuvant treatment. The OS is known as the period from the beginning of randomization to death from any cause. The PFS is known as the period from the beginning of randomization to tumorigenesis, progression, or death from any cause. We performed an NMA through random and fixed effect models for direct and indirect treatment comparison for each outcome (28). In order to assess PFS and OS, contrast-based analysis was used, with estimated differences in log HR and standard error computed using reported HRs and CIs (29). The HR and 95% credible interval (CI) were used to represent relative treatment effects (28). AEs at high grade (grade 3-4) were reported with odds ratios (ORs) and 95% CI based on the available raw data from the selected studies. The connection of the treatment networks in terms of OS, PFS, and AEs was depicted using network plots. I2 was used to assess heterogeneity when multiple trials were available for a given comparison. All statistical analyses were carried out using R software (Version 4.0.4) and STATA (Version 15.0); P < 0.05 was deemed statistically significant. In addition, the software Revman version 5.3 (Cochrane, UK) is used to describe the deviation risk summary, and deviation risk diagram. The current NMA did not need ethical approval since it just collected and evaluated data from previously published studies.

## Results

### Study Selection

**Figure 1:**
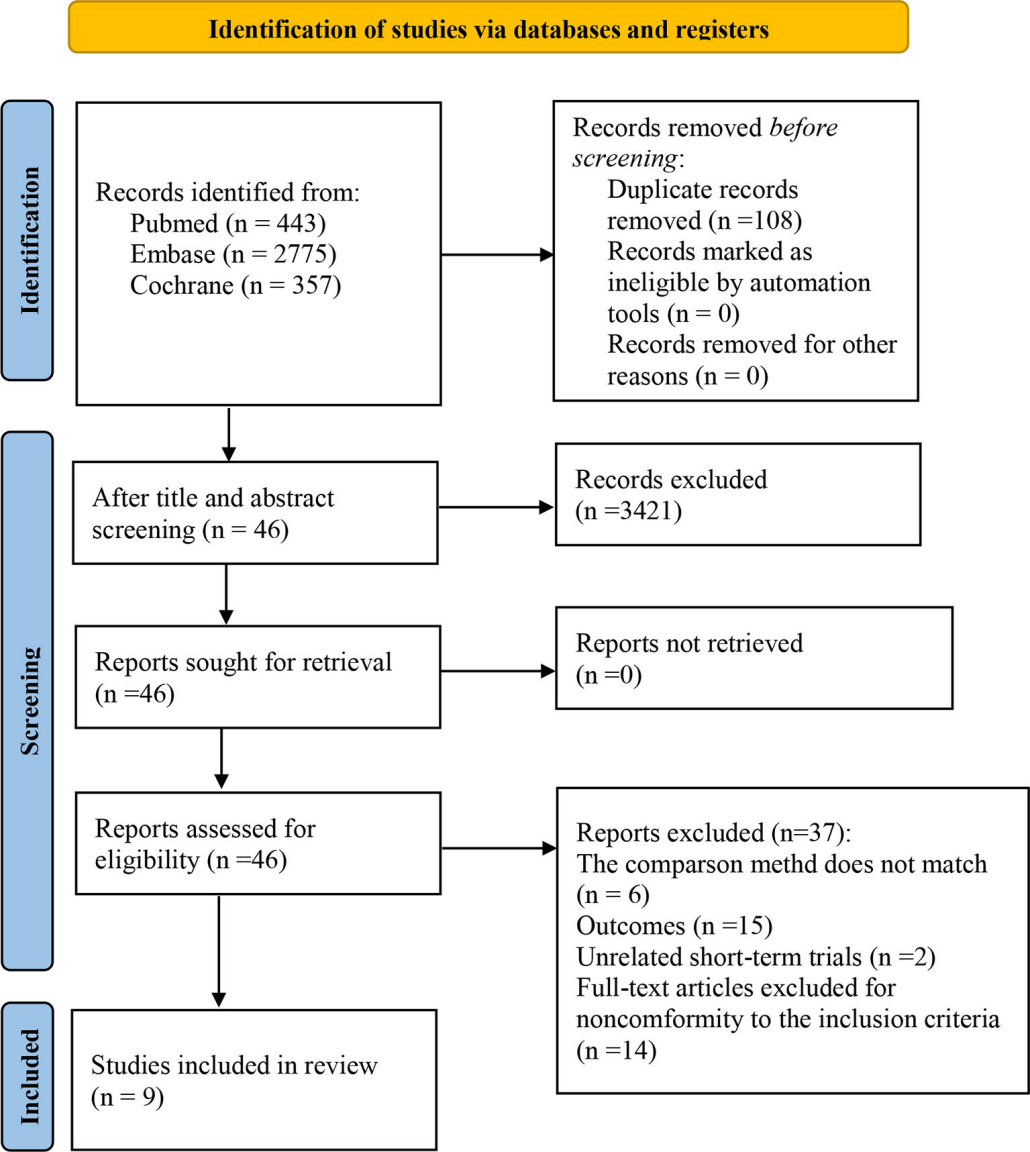
PRISMA flow chart of the study selection process.

We selected 3575 studies in total. Following an eligibility evaluation and a thorough analysis of the full text, 11 studies including 8 different forms of treatment were examined (17–19, 21, 30–36).4 out of 11 trials (18, 19, 21, 30) were short-term outcomes of the clinical trial (trial registration: NCT01844505/NCT01721772/NCT00324155, respectively), therefore we excluded them and chose the latest trials with longer follow-up time. The latest clinical trial (trial registration: NCT01844505) did not report AEs, and the other (NCT00324155) did not report PFS and AEs (related to therapeutic drugs). Consequently, two short-term trials were retained (18, 21). Overall, nine eligible randomized trials were included in this study (18, 21, 31, 36). **Figure 1** depicts the PRISMA flow diagram of study selection.

### Characteristics of Included Trials

Trials were conducted with adult patients with no prior systemic therapies, and unresectable or metastatic histologically confirmed stage III or IV wild-type BRAF melanoma. The nine trials evaluated in the study were conducted between 2010 and 2022 and involved a total of 3077 patients. The largest sample size was 945, while the smallest was 72. The median age was 54-67 years, and the percentage of male patients across the trials ranged from 45% to 74.3%. All patients were randomly allocated to receive one of eight treatment approaches: ipilimumab+dacarbazine (IPI+DTIC), dacarbazine (DTIC), ipilimumab (IPI), trametinib (TRAM), ipilimumab+gp100 (IPI+gp100), gp100, nivolumab (NIVO), nivolumab+ipilimumab (NIVO+IPI). Eight of 9 studies were multicentre trials, and 4 trials reported regions of involved centers. Two short-term trials are included in the 9 trials, which were short-term outcomes of the clinical trial (trial registration: NCT01844505/NCT00324155, respectively) (18, 21). Seven studies were double-blind, and two studies were open‐label. The characteristics of all trials are presented in **Tables 1**, **2**.

**Table 1:** Characteristics of randomized controlled trials included in the NMA.

Trial/publish year	Clinical Trials. gov number	Author country	Type of studies	Treatment 1	Treatment 2	Treatment 3	Multicenter	Region	Minimum follow-up (month)	Outcome
Postow MA/2015 (24)	NCT01927419	USA	RCT	NIVO+IPI	IPI	NA	NR	USA France	11	PFS ORR AEs
Maio M/2015 (25)	NCT00324155	Italy	RCT	IPI+DTIC	DTIC	NA	YES	NR	60	OS ORR AEs
Flaherty KT/2012 (26)	NCT01245062	USA	RandomizedControlled Open-label	TRAM	DTIC	NA	YES	NR	NR	PFS OS ORR AEs
Robert C/2011 (20)	NCT00324155	France	RCT	IPI+DTIC	DTIC	NA	YES	NR	54	PFS OS ORR AEs
Hersh EM/2011 (27)	NCT00050102	USA	RandomizedOpen-label	IPI	IPI+DTIC	NA	YES	NR	NR	ORR OS AEs
Hodi FS/2010 (28)	NCT00094653	USA	RCT	IPI+gp100	IPI	IPI	YES	North America South America Europe Africa	NR	PFS OS ORR AEs
Robert C/2020 (29)	NCT01721772	France	RCT	NIVO	DTIC	NA	YES	Europe Canada Israel Australia South America	60	OS PFS ORR AEs
Larkin J/2019 (23)	NCT01844505	United Kingdom	RCT	NIVO+IPI	NIVO	IPI	YES	Australia Europe Israel New Zealand North America	60	PFS OS ORR AEs
Wolchok JD/2022 (30)	NCT01844505	USA	RCT	NIVO+IPI	NIVO	IPI	YES	Australia Europe Israel New Zealand North America	77	PFS OS ORR MSS

NIVO, Nivolumab IPI, Ipilimumab; DTIC, dacarbazine; TRAM, trametinib; RCT, Randomized controlled trial; NA, not applicable; NMA, network meta-analysis; NR, not reported; PFS, progression-free survival; ORR, objective response rate; OS, overall survival; AEs, adverse event; MSS, Melanoma-specific survival.

**Table 2:** Characteristics of patients in randomized controlled trials included in network meta-analyses.

Trial/publish year	Intervention	N	Median age(range)	Male, n (%)	Race, n (%)				Metastasis stage — no. (%)			
					White	Caucasian	Black	Hispanic	M0	M1a	M1b	M1c
Postow MA/2015 (24)	NIVO+IPI	95	64 (27, 87)	63 (66.3)	NR	NR	NR	NR	8(8.4)	15(15.8)	27(28.4)	44(46.3)
	IPI	47	67 (31, 80)	32 (68.1)	NR	NR	NR	NR	5(10.6)	8(17.0)	12(25.5)	21(44.7)
Maio M/2015 (25)	IPI+DTIC (Patients Alive≥5 Years)	40	57.5(33,87)	25(62.5)	NR	NR	NR	NR	3(7.5)	8(20.0)	11(27.5)	18(45.0)
	DTIC (Patients Alive≥5 Years)	20	61.0(31,76)	9(45.0)	NR	NR	NR	NR	1(5.0)	10(50.0)	4(20.0)	5(25.0)
Flaherty KT/2012 (26)	TRAM	214	55(23,85)	120(56.0)	214(100)	NR	NR	NR	NR	24(11.2)	35(16.4)	144(67.3)
	DTIC	108	54(21,77)	53(49.0)	108(100)	NR	NR	NR	NR	15(13.9)	22(20.4)	63(58.3)
Robert C/2011 (20)	IPI+DTIC	250	57.5	152(60.8)	NR	NR	NR	NR	6(2.4)	37(14.8)	64(25.6)	143(57.2)
	DTIC	252	56.4	149(59.1)	NR	NR	NR	NR	8(3.2)	43(17.1)	62(24.6)	139(55.2)
Hersh EM/2011 (27)	IPI	37	66.0(25,82)	21(56.8)	NR	34(91.9)	1(2.7)	2(5.4)	NR	8(21.6)	8(21.6)	21(56.8)
	IPI+DTIC	35	60.0(27,82)	26(74.3)	NR	31(88.6)	2(5.7)	2(5.7)	NR	6(17.1)	12(34.3)	16(45.7)
Hodi FS/2010 (28)	IPI+gp100	403	55.6	247(61.3)	NR	NR	NR	NR	5(1.2)	37(9.2)	76(18.9)	285(70.7)
	IPI	137	56.8	81(59.1)	NR	NR	NR	NR	1(0.7)	14(10.2)	22(16.1)	100(73.0)
	gp100	136	57.4	73(53.7)	NR	NR	NR	NR	4(2.9)	11(8.1)	23(16.9)	98(72.1)
Robert C/2020 (29)	NIVO	210	64(18,86)	121(57.6)	NR	NR	NR	NR	82(39.0)			111(52.9)
	DTIC	208	66(26,87)	125(60.1)	NR	NR	NR	NR	81(38.9)			87(41.8)
Larkin J/2019 (23)	NIVO+IPI	314	61(18,88)	206(65.6)	NR	NR	NR	NR	133(42.4)			181(57.6)
	NIVO	316	60(25,90)	202(63.9)	NR	NR	NR	NR	132(41.8)			184(58.2)
	IPI	315	62(18,89)	202(64.1)	NR	NR	NR	NR	132(41.9)			183(58.1)
Wolchok JD/2022 (30)	NIVO+IPI	314	61(18,88)	206(65.6)	NR	NR	NR	NR	133(42.4)			181(57.6)
	NIVO	316	60(25,90)	202(63.9)	NR	NR	NR	NR	132(41.8)			184(58.2)
	IPI	315	62(18,89)	202(64.1)	NR	NR	NR	NR	132(41.9)			183(58.1)

NIVO, Nivolumab; IPI, Ipilimumab; DTIC, dacarbazine; TRAM, trametinib; RCT, Randomized controlled trial; NA, not applicable; NMA, network meta-analysis; NR, not reported; PFS, progression-free survival; ORR, objective response rate; OS, overall survival; AEs, adverse event; MSS, Melanoma-specific survival.

### Quality Assessment of the Included Studies

**Figure 2:**
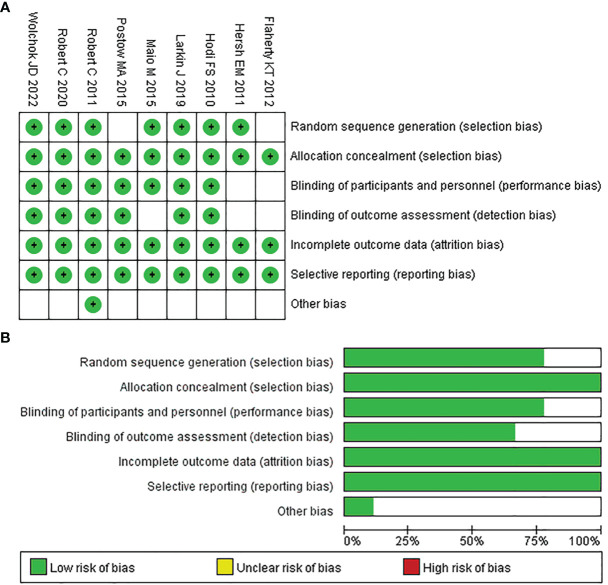
Quality assessment of included trials in the NMA. Risk of bias summary **(A)** and Risk of bias graph **(B)**.

After the quality evaluation by the tool of Cochrane Collaboration, we found that all included studies did not show obvious publication bias in this NMA. As seven of the nine papers provided adequate procedures for generating random sequences, their selection bias was rated as “low risk.” Also because the remaining studies only mentioned “random,” the selection bias of two of them was rated as “unclear risk.” Since all of the studies reported the processes used for allocation concealment, their bias was classified as “unclear risk.” Seven studies indicated participant and personnel blinding, hence their bias was rated as “low risk.” Two studies did not specify the blinding of participants and personnel, so their bias was assessed as “unclear risk”. Six studies mentioned the outcome assessment blinding; thus, their bias was rated as “low risk”; and the risk of bias in the remaining studies was regarded as “unclear risk”. For incomplete outcome data, all studies were rated as “low risk”. Because all of the studies provided the results mentioned in the method section, the reporting bias was rated as “low risk.” The detailed assessment results are shown in **Figures 2A** and **B**.

### NMA

**Figure 3:**
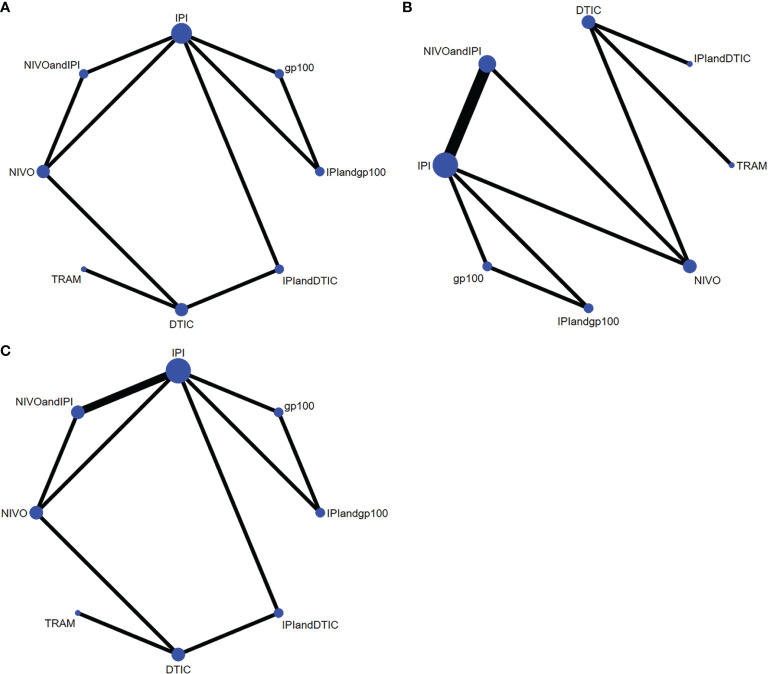
Network constructions for comparisons in OS, PFS and AEs: **(A)** network constructions for OS, **(B)** network constructions for PFS, **(C)** network constructions for AEs.

In terms of OS, PFS, and AEs, the networks of eligible comparisons were graphically displayed in network plots (**Figures 3A–C**).

### Efficacy

Among the eight types of therapy, three showed significant benefits: NIVO+IPI, NIVO, and TRAM. As demonstrated by the predictions and confidence intervals of OS and PFS, no heterogeneity was present (I2 = 0%).

### Overall Survival

**Figure 4:**
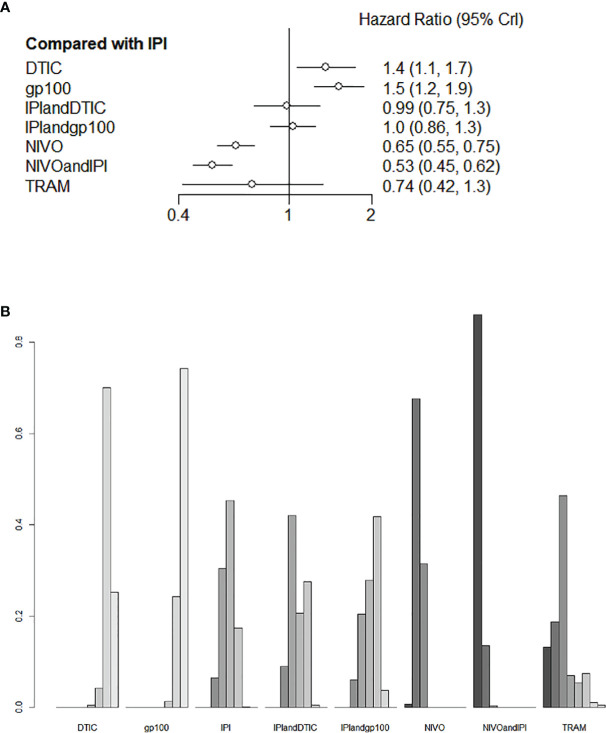
Direct and indirect comparisons for OS **(A, B)** among IPI+DTIC, IPI+gp100, DTIC, IPI, gp100, NIVO, NIVO+IPI, TRAM.

Six of the nine trials with a total of 2621 individuals had OS data available. IPI+DTIC considerably enhanced OS when compared to IPI, NIVO+IPI, NIVO, TRAM, and IPI+IVO (HR 0.53, 95% CI 0.45-0.62; HR 0.65, 95% CI 0.55-0.75; HR 0.74, 95% CI 0.42-1.1; and HR 0.99, 95% CI 0.75-1.3, respectively; **Figure 4A**). Based on the treatments ranking analysis, NIVO+IPI had the highest potential of providing the best OS (**Figure 4B**). When comparing each intervention, it was found that NIVO was correlated with poorer OS than NIVO+IPI (HR 1.22, 95% CI 1.03-1.44; **Figure S1**). Moreover, NIVO was significantly more effective for promoting OS (HR 0.87, 95% CI 0.49-1.53; **Figure S1**) than TRAM. When compared to TRAM, NIVO+IPI considerably enhanced OS (HR 0.72, 95% CI 0.4-1.28; **Figure S1**). Interestingly, gp100 was not conducive to OS compared with any other therapies. Additionally, we found no statistically significant difference between direct and indirect comparisons (P > 0.05). The heterogeneity of this analysis was low (I2 = 0%). All outcomes of comparisons for OS are presented in **Figure 4** and **Figure S1**.

### Progression-Free Survival

**Figure 5:**
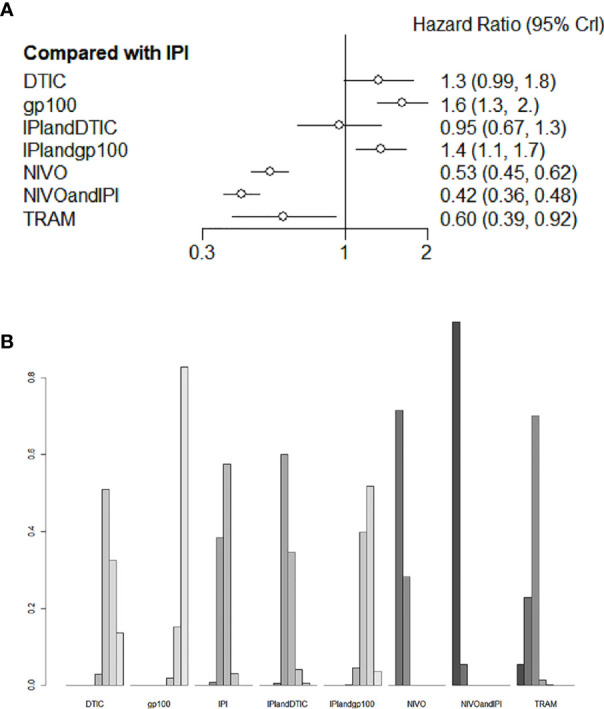
Direct and indirect comparisons for PFS **(A, B)** among IPI+DTIC, IPI+gp100, DTIC, IPI, gp100, NIVO, NIVO+IPI, TRAM.

Six studies evaluated eight different agents, which contributed to the PFS analysis. Three treatments clearly stood better than IPI (**Figure 5A**): NIVO+IPI (HR 0.42, 95% CI 0.36-0.48), NIVO (HR 0.53, 95% CI 0.45-0.62), and TRAM (HR 0.60, 95% CI 0.39-0.92). Based on the treatment ranking analysis, NIVO+IPI had the highest potential of giving the best PFS (**Figure 5B**). Compared with TRAM, NIVO+IPI, and NIVO significantly improved PFS (HR 0.7, 95% CI 0.45-1.08; and HR 0.89, 95% CI 0.59-1.33, respectively; **Figure S2**). NIVO+IPI was significantly more effective for promoting PFS (HR 0.79, 95% CI 0.67-0.92; **Figure S2**) than NIVO. Interestingly, gp100 was not conducive to PFS compared with any other therapy. Moreover, we also found that there was no statistical difference between direct comparison and indirect comparison (P > 0.05). The heterogeneity of this analysis was low (I2 = 0%). **Figure 5** and **Figure S2** provide the full comparative PFS results.

### Adverse Events

**Figure 6:**
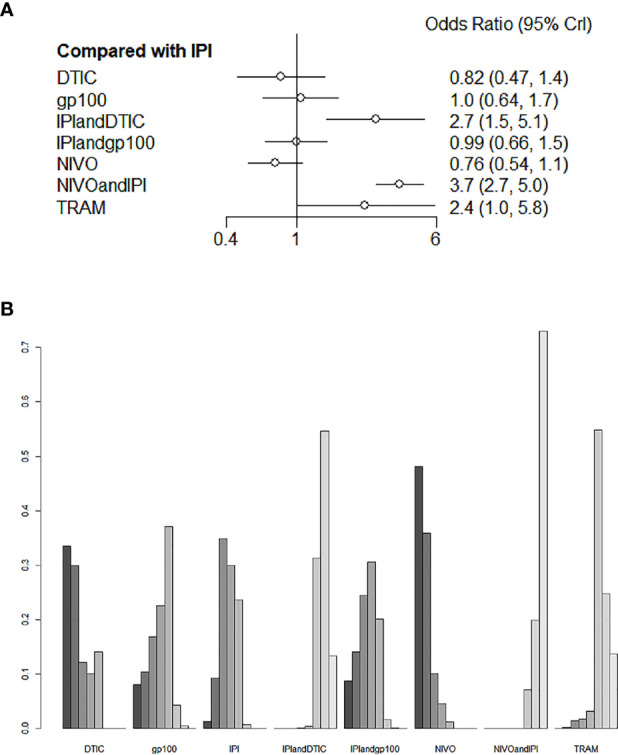
Direct and indirect comparisons for AEs **(A, B)** among IPI+DTIC, IPI+gp100, DTIC, IPI, gp100, NIVO, NIVO+IPI, TRAM.

For the varied AE outcomes, an NMA testing of seven different agents was performed (grade 3 or 4). Compared with IPI, NIVO, DTIC, and IPI+gp100 had a lower risk of adverse reactions (HR 0.76, 95% CI 0.54-1.1; HR 0.82, 95% CI 0.47-1.4; and HR 0.99, 95% CI 0.66-1.5, respectively; **Figure 6A**); the risk of adverse reactions caused by IPI+DTIC and TRAM was relatively high (HR 2.7, 95% CI 1.5-5.1; and HR 2.4, 95% CI 1.0-5.8, respectively; **Figure 6A**); and NIVO+IPI showed the highest potential of adverse reactions (HR 3.7, 95% CI 2.7-5.0, **Figure 6A**). Based on the treatment ranking analysis, NIVO had the lowest risk of AEs (grade 3 or 4; **Figure 6B**). Compared with IPI+DTIC, IPI and DTIC exhibited a lower probability of adverse events (HR 0.37, 95% CI 0.2-0.69; and HR 0.3, 95% CI 0.21-0.43, respectively; **Figure S3**). On the other hand, IPI+gp100 had a higher probability of adverse events than IPI and gp100 (HR 1.01, 95% CI 0.68-1.51; and HR 1.06, 95% CI 0.71-1.57, respectively; **Figure S3**). The heterogeneity of this analysis was low (I2 = 0%). All AEs of grade 3 or 4 are presented in **Figure 6** and **Figure S3**.

## Discussion

This meta-analysis explores the most effective and safest adjuvant treatments for unresectable advanced or metastatic melanoma, based on the drugs currently available on the market. We conducted a thorough search for qualifying RCTs, critically evaluated trial quality, meticulously synthesized trial data, and finally, classified treatments based on the efficacy and safety demonstrated in randomized clinical trials. The network method attempts to prevent the lack of direct comparison across several available options, particularly the comparison of checkpoint inhibitors to targeted therapies as well as checkpoint inhibitors Therefore, we conducted NMA to evaluate their efficacy and safety indirectly. This method yielded intriguing findings.

Our findings revealed that NIVO+IPI was superior to other therapies in terms of increasing OS and PFS in advanced melanoma patients. The combination of NIVO and IPI was considered to have complimentary benefits in the treatment of metastatic melanoma, and our findings were consistent with previous research (37). Although single-agent NIVO was ranked lower than NIVO+IPI, it might still offer more advantages in terms of OS and PFS than any other treatments. Additionally, TRAM (an investigational hot spot in targeted therapy) is a specific allosteric inhibitor of MEK1/2, and many trials with supportive preclinical evidence confirmed its efficacy in non-V600 mutant melanomas (38–40). In addition to NIVO+IPI and NIVO, TRAM appeared to be more efficacious than other treatments in improving OS, and PFS. Thus, it is reasonable to believe that NIVO+IPI, NIVO, and TRAM targeted therapies remarkably have improved OS and PFS in patients with unresectable advanced or metastatic melanoma.

Among the authorized therapy options at the time of this study, NIVO+IPI and NIVO had the longest follow-up duration. Long-term survival studies have shown a considerable improvement in OS with NIVO alone or NIVO+IPI compared to IPI alone. The median OS of NIVO+IPI was around twice as long as that of NIVO alone, showing that the combination’s survival rate was much higher than that of NIVO alone (36). Similarly, for melanoma patients with BRAF mutations, NIVO-containing regimens still outperformed IPI alone in terms of survival (21, 31, 36). Overall, the NIVO+IPI response characteristics detected in this investigation were similar to previously reported results (41, 42). Despite checkpoint inhibitors’ dominance, TRAM still improved PFS and OS among metastatic melanoma patients with BRAF V600E or V600K mutation. Therefore, TRAM may be an alternate option for BRAF wild-type or BRAF-mutated patients.

Although adjuvant treatments have offered significant benefits for advanced melanoma, they still have some limitations. Since chemotherapy cannot differentiate between cancer and healthy cell types, it will damage both (43). Similarly, high radiation doses can also damage surrounding healthy tissues. As hormone treatment alters hormone levels and function, it may cause unwanted side effects including organ dysfunction (44). In addition, the combination of BRAFi and MEKi has shown clinical effectiveness and long-term disease control in metastatic melanoma. However, there may be a drug resistance mechanism during therapy, and around 15% of patients are intolerant to treatment (45). Ipilimumab stimulates T-cell proliferation, which can result in immune-related side effects such as dermatitis, endocrinopathy, and hepatitis, as well as other side effects like pruritus, fatigue, and colitis (46–48). Our current study highlighted grade 3 and 4 AEs associated with different immune checkpoint inhibitors and targeted therapies. The NIVO group showed the lowest chance of developing grade 3/4 AEs, followed by DTIC. Most notably, NIVO+IPI had the greatest risk of grade 3 or 4 AEs. It indicates that NIVO+IPI was highly efficacious while also having a significant level of toxicity. TRAM also showed a relatively higher probability of grade 3 or 4 AEs. Another study discovered that the high toxicity of checkpoint inhibitors made the development of combination therapy problematic (49). In this study, the combination of IPI and DTIC had high toxicity, but the combination of IPI and gp100 showed a relatively lower rate of grade 3 or 4 AEs. IPI+DTIC and IPI+gp100 were more effective in improving OS/PFS over monotherapy (IPI, DTIC, IPI, gp100). Therefore, we hypothesized that IPI+gp100, rather than IPI+DTIC, appeared to be more suitable for long-term therapy in patients with advanced melanoma.

This meta-analysis investigated the optimum adjuvant treatment for advanced melanoma and provided clinical suggestions on different administration regimens. As in our research, NIVO+IPI was the most effective in extending the survival of advanced melanoma patients, although it was associated with an excessive number of AEs. As the result, NIVO+IPI treatment should be carefully considered for advanced melanoma patients with poor physical conditions. Similarly, the side effects of TRAM also limit its use. NIVO dramatically increased the survival of patients with advanced melanoma, ranked second only to NIVO+IPI. Simultaneously, across all treatment modalities, NIVO showed the lowest rate of grade 3 or 4 AEs, and its safety profile was manageable. Undoubtedly, NIVO is the best appropriate treatment in this study for patients with unresectable advanced or metastatic melanoma. This conclusion is consistent with previous research results (50). Compared with monotherapy, combination therapy was more beneficial to improve OS and PFS for advanced melanoma patients. IPI+gp100 ranked below IPI+DTIC and NIVO+IPI with concerning OS/PFS, but IPI+gp100 had a lower rate of grade 3 or 4 AEs compared to other treatments (except NIVO). More importantly, additional innovations need to be explored in the future to alleviate the toxicity associated with combination therapy.

However, this analysis has several limitations. Firstly, we were unable to obtain detailed individual patient data, which limited our ability to evaluate outcomes and patient characteristics.Secondly, we conducted our data analysis on a relatively small number of included RCTs. Thirdly, we did not analyze patients with BRAF mutation-positive tumors, because subgroup analysis could not be performed in the metadata. Fourthly, two of the trials included in this study were open-ended, which might introduce unintentional bias. Furthermore, due to the limitation of just including level 3 or 4 AES in this analysis, some outcomes might be inconsistent with reality. According to our results, NIVO had a high therapeutic effect and the lowest toxicity; nevertheless, large-scale prospective studies were necessary to provide credible evidence.

Despite all of the shortcomings described above, we can properly compare several adjuvant treatments and propose the best therapy for advanced melanoma. At the moment, single-agent NIVO is a suitable option for patients with unresectable advanced or metastatic melanoma. Longer follow-up in those adjuvant treatments, combined with further investigation of combination treatments, may improve outcomes in advanced melanoma.

## Conclusions

In conclusion, NIVO is the best adjuvant therapy with a promising profile for patients with unresectable advanced or metastatic melanoma. This study offered evidence for the comparison among these adjuvant treatments. NIVO+IPI ranked first in efficacy but had the highest toxicity. TRAM ranked third in efficacy but had high toxicity. Combination therapy is more successful in treating unresectable advanced or metastatic melanoma, although it is associated with a higher risk of adverse events. Conversely, IPI+gp100 had a lower rate of grade 3 or 4 AEs than other treatments (except NIVO). These results might have a significant impact on the individualized therapy of patients with advanced melanoma.

However, there are several limitations to this overview. First, we may be missing some information because only SRs published in English are included. Furthermore, the sample size of this study was relatively small. Second, we could not obtain detailed data from each patient, which limited the evaluation of outcomes. Third, we were unable to conduct a subgroup analysis of BRAF mutation-positive tumors patients. Finally, the subjective assessment of the authors may affect the outcome of the quality evaluation process. In the future, for the treatment of patients with advanced melanoma, clinicians must consider the efficacy and safety of monotherapy and combination therapy, and the patients’ physical status. MBGs, nanotechnology, and tannic acid-incorporated medical applications have bright prospects in the treatment of advanced melanoma. More new schemes about adjuvant treatments are necessary to provide stronger evidence for definitive conclusions.

## Data Availability Statement

The raw data supporting the conclusions of this article will be made available by the authors, without undue reservation.

## Author Contributions

MJ, Yc, JS, and XZ contributed equally to the work. MJ, Yc contributed to the initial design and drafting of the research. MJ, JS and XZ participated in the drafting process process and analyzed the data. FY evaluated the data. BZ and JZ participated in article revision. MX and MC supervised the study. All authors contributed to the article and approved the submitted version.

## Funding

The study was funded by the Sichuan Provincial Administration of Traditional Chinese Medicine (Grant No. H2021076).

## Conflict of Interest

The authors declare that the research was conducted in the absence of any commercial or financial relationships that could be construed as a potential conflict of interest.

## Publisher’s Note

All claims expressed in this article are solely those of the authors and do not necessarily represent those of their affiliated organizations, or those of the publisher, the editors and the reviewers. Any product that may be evaluated in this article, or claim that may be made by its manufacturer, is not guaranteed or endorsed by the publisher.

## Abbreviations

OS, overall survival; PFS, progression-free survival; AEs, adverse effects; RCT, randomized clinical trial; NMA, network meta-analysis; CTLA-4, cytotoxic T lymphocyte-associated protein 4; PD-1, programmed cell death protein 1; PD-L1, programmed cell death ligand 1; FDA, the United States Food and Drug Administration; NCT, National Clinical Trial; CIs, confidence intervals; IPI+DTIC, ipilimumab+dacarbazine; DTIC, dacarbazine; IPI, ipilimumab; TRAM, trametinib; IPI+gp100, ipilimumab+gp100; NIVO, nivolumab; NIVO+IPI, nivolumab+ipilimumab; MBGs, mesoporous bioactive glasses.
