# Supplementary material for: Adjuvant Treatments of Adult Melanoma: A Systematic Review and Network Meta-Analysis
Source: Front Oncol. 2022 Jun 17;12:926242. doi: 10.3389/fonc.2022.926242 (PMC9247312; doi:10.3389/fonc.2022.926242)
Supplement: Supplementary file 1 [file DataSheet_1.pdf]

DTIC	1.11 (0.81, 1.52)	0.73 (0.57, 0.93)	0.72 (0.6, 0.86)	0.76 (0.56, 1.03)	0.47 (0.38, 0.58)	0.39 (0.3, 0.5)	0.54 (0.32, 0.92)
0.9 (0.66, 1.23)	gp100	0.66 (0.54, 0.8)	0.65 (0.46, 0.91)	0.68 (0.57, 0.82)	0.42 (0.33, 0.55)	0.35 (0.27, 0.45)	0.49 (0.26, 0.9)
1.37 (1.07, 1.74)	1.52 (1.24, 1.87)	IPI	0.99 (0.75, 1.3)	1.04 (0.86, 1.25)	0.65 (0.55, 0.75)	0.53 (0.45, 0.62)	0.74 (0.42, 1.33)
1.39 (1.16, 1.66)	1.54 (1.1, 2.16)	1.02 (0.77, 1.33)	IPIandDTIC	1.05 (0.75, 1.46)	0.65 (0.51, 0.84)	0.54 (0.4, 0.72)	0.75 (0.43, 1.31)
1.32 (0.97, 1.79)	1.47 (1.22, 1.76)	0.96 (0.8, 1.16)	0.95 (0.68, 1.32)	IPIandgp100	0.62 (0.49, 0.79)	0.51 (0.4, 0.65)	0.71 (0.39, 1.32)
2.12 (1.72, 2.61)	2.36 (1.83, 3.04)	1.55 (1.33, 1.81)	1.53 (1.19, 1.97)	1.61 (1.26, 2.05)	NIVO	0.82 (0.69, 0.97)	1.15 (0.65, 2.03)
2.58 (2, 3.36)	2.87 (2.22, 3.72)	1.89 (1.61, 2.23)	1.86 (1.39, 2.5)	1.96 (1.53, 2.51)	1.22 (1.03, 1.44)	NIVOandIPI	1.4 (0.78, 2.52)
1.85 (1.09, 3.13)	2.06 (1.11, 3.8)	1.35 (0.75, 2.41)	1.33 (0.76, 2.33)	1.4 (0.76, 2.58)	0.87 (0.49, 1.53)	0.72 (0.4, 1.28)	TRAM

Figure S1 Head-to-head comparisons for OS in NMA
