# Supplementary material for: Adjuvant Treatments of Adult Melanoma: A Systematic Review and Network Meta-Analysis
Source: Front Oncol. 2022 Jun 17;12:926242. doi: 10.3389/fonc.2022.926242 (PMC9247312; doi:10.3389/fonc.2022.926242)
Supplement: Supplementary file 2 [file DataSheet_2.pdf]

DTIC	1.22 (0.85, 1.75)	0.76 (0.57, 1.01)	0.72 (0.59, 0.87)	1.02 (0.71, 1.46)	0.4 (0.31, 0.51)	0.32 (0.24, 0.42)	0.45 (0.33, 0.62)
0.82 (0.57, 1.18)	gp100	0.62 (0.5, 0.76)	0.59 (0.39, 0.89)	0.84 (0.68, 1.03)	0.33 (0.25, 0.43)	0.26 (0.2, 0.33)	0.37 (0.23, 0.6)
1.32 (0.99, 1.77)	1.61 (1.31, 1.99)	IPI	0.95 (0.67, 1.35)	1.35 (1.1, 1.66)	0.53 (0.45, 0.62)	0.42 (0.36, 0.48)	0.6 (0.39, 0.92)
1.39 (1.15, 1.68)	1.69 (1.12, 2.55)	1.05 (0.74, 1.49)	IPlandDTIC	1.42 (0.95, 2.13)	0.56 (0.41, 0.76)	0.44 (0.31, 0.62)	0.63 (0.43, 0.91)
0.98 (0.68, 1.4)	1.19 (0.97, 1.47)	0.74 (0.6, 0.91)	0.71 (0.47, 1.06)	IPlandgp100	0.39 (0.3, 0.51)	0.31 (0.24, 0.4)	0.44 (0.27, 0.71)
2.5 (1.95, 3.2)	3.05 (2.35, 3.96)	1.89 (1.62, 2.2)	1.8 (1.32, 2.46)	2.55 (1.97, 3.31)	NIVO	0.79 (0.67, 0.92)	1.13 (0.75, 1.69)
3.17 (2.37, 4.24)	3.87 (2.99, 5.01)	2.4 (2.07, 2.78)	2.29 (1.61, 3.23)	3.24 (2.51, 4.18)	1.27 (1.08, 1.48)	NIVOandIPI	1.43 (0.93, 2.21)
2.22 (1.61, 3.06)	2.71 (1.67, 4.39)	1.68 (1.09, 2.59)	1.6 (1.1, 2.33)	2.27 (1.4, 3.67)	0.89 (0.59, 1.33)	0.7 (0.45, 1.08)	TRAM

Figure S2 Head-to-head comparisons for PFS in NMA
