# Supplementary material for: Adjuvant Treatments of Adult Melanoma: A Systematic Review and Network Meta-Analysis
Source: Front Oncol. 2022 Jun 17;12:926242. doi: 10.3389/fonc.2022.926242 (PMC9247312; doi:10.3389/fonc.2022.926242)
Supplement: Supplementary file 3 [file DataSheet_3.pdf]

DTIC	1.28 (0.61, 2.68)	1.22 (0.7, 2.14)	3.33 (2.33, 4.8)	1.21 (0.61, 2.41)	0.93 (0.58, 1.5)	4.52 (2.57, 7.99)	2.89 (1.49, 5.86)
0.78 (0.37, 1.64)	gp100	0.96 (0.59, 1.55)	2.61 (1.19, 5.8)	0.94 (0.64, 1.41)	0.72 (0.4, 1.31)	3.53 (1.99, 6.28)	2.26 (0.83, 6.36)
0.82 (0.47, 1.42)	1.05 (0.64, 1.69)	IPI	2.73 (1.46, 5.1)	0.99 (0.66, 1.47)	0.76 (0.54, 1.07)	3.7 (2.74, 5.04)	2.37 (1, 5.82)
0.3 (0.21, 0.43)	0.38 (0.17, 0.84)	0.37 (0.2, 0.69)	IPIandDTIC	0.36 (0.17, 0.76)	0.28 (0.16, 0.49)	1.36 (0.72, 2.57)	0.87 (0.41, 1.91)
0.83 (0.41, 1.63)	1.06 (0.71, 1.57)	1.01 (0.68, 1.51)	2.76 (1.31, 5.84)	IPIandgp100	0.77 (0.45, 1.3)	3.74 (2.26, 6.19)	2.4 (0.91, 6.42)
1.08 (0.67, 1.73)	1.38 (0.76, 2.49)	1.32 (0.94, 1.85)	3.59 (2.03, 6.34)	1.3 (0.77, 2.21)	NIVO	4.86 (3.49, 6.85)	3.11 (1.37, 7.31)
0.22 (0.13, 0.39)	0.28 (0.16, 0.5)	0.27 (0.2, 0.37)	0.74 (0.39, 1.4)	0.27 (0.16, 0.44)	0.21 (0.15, 0.29)	NIVOandIPI	0.64 (0.27, 1.58)
0.35 (0.17, 0.67)	0.44 (0.16, 1.21)	0.42 (0.17, 1)	1.15 (0.52, 2.45)	0.42 (0.16, 1.1)	0.32 (0.14, 0.73)	1.56 (0.63, 3.76)	TRAM

Figure S3 Head-to-head comparisons for AEs in NMA
