# Supplementary material for: Adjuvant Treatments of Adult Melanoma: A Systematic Review and Network Meta-Analysis
Source: Front Oncol. 2022 Jun 17;12:926242. doi: 10.3389/fonc.2022.926242 (PMC9247312; doi:10.3389/fonc.2022.926242)
Supplement: Supplementary file 4 [file Table_1.docx]

| **Table S1 Search Strategy** | | |
| --- | --- | --- |
| **PubMed** | | |
| **Step** | **Query** | **Items found** |
| **#1:** | **Search**: "Melanoma"[**MeSH Terms**] OR "Melanomas"[Title/Abstract] OR "Malignant Melanoma"[Title/Abstract] OR "Malignant Melanomas"[Title/Abstract] OR "Melanoma, Malignant"[Title/Abstract] OR "Melanomas, Malignant"[Title/Abstract] | **112144** |
| **#2:** | **Search**: "Chemotherapy, Adjuvant"[Mesh Terms] OR "Drug Therapy, Adjuvant"[Title/Abstract] OR "Adjuvant Chemotherapy"[Title/Abstract] OR "Adjuvant Drug Therapy"[Title/Abstract] OR "Molecular Targeted Therapy"[Mesh] OR "Molecular Targeted Therapies"[Title/Abstract] OR "Targeted Therapy, Molecular"[Title/Abstract] OR "Therapy, Molecular Targeted"[Title/Abstract] OR "Targeted Molecular Therapy"[Title/Abstract] OR "Molecular Therapy, Targeted"[Title/Abstract] OR "Targeted Molecular Therapies"[Title/Abstract] OR "Therapy, Targeted Molecular"[Title/Abstract] OR "Vemurafenib"[Mesh] OR "PLX4032"[Title/Abstract] OR "PLX 4032"[Title/Abstract] OR "RG7204"[Title/Abstract] OR "RG-7204"[Title/Abstract] OR "RG 7204"[Title/Abstract] OR "Zelboraf"[Title/Abstract] OR "R05185426"[Title/Abstract] OR "dabrafenib" [Supplementary Concept] OR "GSK 2118436"[Title/Abstract] OR "GSK2118436"[Title/Abstract] OR "GSK-2118436"[Title/Abstract] OR "trametinib" [Supplementary Concept] OR "JTP 74057"[Title/Abstract] OR "JTP74057"[Title/Abstract] OR "JTP-74057"[Title/Abstract] OR "GSK 1120212"[Title/Abstract] OR "GSK1120212[Title/Abstract]" OR "GSK-1120212"[Title/Abstract] OR "encorafenib" [Supplementary Concept] OR "LGX818"[Title/Abstract] OR "Braftovi"[Title/Abstract] OR "binimetinib" [Supplementary Concept] OR "Mektovi"[Title/Abstract] OR "MEK162"[Title/Abstract] OR "Ipilimumab"[Mesh] OR "Anti-CTLA-4 MAb Ipilimumab"[Title/Abstract] OR "Anti CTLA 4 MAb Ipilimumab"[Title/Abstract] OR "Ipilimumab, Anti-CTLA-4 MAb"[Title/Abstract] OR "Yervoy"[Title/Abstract] OR "MDX 010"[Title/Abstract] OR "MDX010"[Title/Abstract] OR "MDX-010"[Title/Abstract] OR "MDX-CTLA-4"[Title/Abstract] OR "MDX CTLA 4"[Title/Abstract] OR "pembrolizumab" [Supplementary Concept] OR "SCH-900475"[Title/Abstract] OR "Keytruda"[Title/Abstract] OR "MK-3475"[Title/Abstract] OR "lambrolizumab"[Title/Abstract] OR "Nivolumab"[Mesh] OR "Opdivo"[Title/Abstract] OR "ONO-4538"[Title/Abstract] OR "ONO 4538"[Title/Abstract] OR "ONO4538"[Title/Abstract] OR "MDX-1106"[Title/Abstract] OR "MDX 1106"[Title/Abstract] OR "MDX1106"[Title/Abstract] OR "BMS-936558"[Title/Abstract] OR "BMS 936558"[Title/Abstract] OR "BMS936558"[Title/Abstract] OR "cemiplimab" [Supplementary Concept] OR "REGN2810"[Title/Abstract] OR "avelumab" [Supplementary Concept] OR "avelumab"[Title/Abstract] OR "MSB0010682"[Title/Abstract] OR "bavencio"[Title/Abstract] OR "MSB0010718C"[Title/Abstract] OR "MSB-0010718C"[Title/Abstract] OR "Immune Checkpoint Inhibitors"[Mesh] OR "Checkpoint Inhibitors, Immune"[Title/Abstract] OR "Immune Checkpoint Inhibitor"[Title/Abstract] OR "Checkpoint Inhibitor, Immune"[Title/Abstract] OR "Immune Checkpoint Blockers"[Title/Abstract] OR "Checkpoint Blockers, Immune"[Title/Abstract] OR "Immune Checkpoint Blockade"[Title/Abstract] OR "Checkpoint Blockade, Immune"[Title/Abstract] OR "Immune Checkpoint Inhibition"[Title/Abstract] OR "Checkpoint Inhibition, Immune"[Title/Abstract] OR "PD-L1 Inhibitors"[Title/Abstract] OR "PD L1 Inhibitors"[Title/Abstract] OR "PD-L1 Inhibitor"[Title/Abstract] OR "PD L1 Inhibitor"Title/Abstract] OR "Programmed Death-Ligand 1 Inhibitors"[Title/Abstract] OR "Programmed Death Ligand 1 Inhibitors"[Title/Abstract] OR "CTLA-4 Inhibitors[Title/Abstract]" OR "CTLA 4 Inhibitors"[Title/Abstract] OR "CTLA-4 Inhibitor"[Title/Abstract] OR "CTLA 4 Inhibitor"[Title/Abstract] OR "Cytotoxic T-Lymphocyte-Associated Protein 4 Inhibitors"[Title/Abstract] OR "Cytotoxic T Lymphocyte Associated Protein 4 Inhibitors"[Title/Abstract] OR"Cytotoxic T-Lymphocyte-Associated Protein 4 Inhibitor"[Title/Abstract] OR "Cytotoxic T Lymphocyte Associated Protein 4 Inhibitor"[Title/Abstract] OR "PD-1 Inhibitors"[Title/Abstract] OR "PD 1 Inhibitors"[Title/Abstract] OR "PD-1 Inhibitor"[Title/Abstract] OR "Inhibitor, PD-1"[Title/Abstract] OR "PD 1 Inhibitor"[Title/Abstract] OR "Programmed Cell Death Protein 1 Inhibitor"[Title/Abstract] OR "Programmed Cell Death Protein 1 Inhibitors"[Title/Abstract] OR "PD-1-PD-L1 Blockade"[Title/Abstract] OR "Blockade, PD-1-PD-L1"[Title/Abstract] OR "PD 1 PD L1 Blockade"[Title/Abstract] OR "cobimetinib" [Supplementary Concept] OR "3,4-difluoro-2-(2-fluoro-4-iodophenylamino)phenyl)(3-hydroxy-3-(piperidin-2-yl)azetidin-1-yl)methanone"[Title/Abstract] OR "Cotellic"[Title/Abstract] OR "GDC-0973"[Title/Abstract] OR "XL518"[Title/Abstract] OR "Cytokine-Induced Killer Cells"[Mesh] OR "Cytokine Induced Killer Cells"[Title/Abstract] OR "Cytokine-Induced Killer Cell"[Title/Abstract] OR "Killer Cell, Cytokine-Induced"[Title/Abstract] OR "Killer Cells, Cytokine-Induced"[Title/Abstract] OR "CIK Cells"[Title/Abstract] OR "CIK Cell"[Title/Abstract] OR "Lymphocyte-Activated Killer Cells"[Title/Abstract] OR "Cell, Lymphocyte-Activated Killer"[Title/Abstract] OR "Cells, Lymphocyte-Activated Killer"[Title/Abstract] OR "Killer Cell, Lymphocyte-Activated"[Title/Abstract] OR "Killer Cells, Lymphocyte-Activated"[Title/Abstract] OR "Lymphocyte Activated Killer Cells"[Title/Abstract] OR "Lymphocyte-Activated Killer Cell"[Title/Abstract] OR "Lymphocyte Activated Killer Cell"[Title/Abstract] OR "talimogene laherparepvec"[Supplementary Concept] OR "T-VEC"[Title/Abstract] | **151723** |
| **#3:** | **Search:** "Review"[Publication Type] OR "Case report"[Publication Type] OR "Letter"[Publication Type] OR "Editoria"[Publication Type] OR "Comment"[Publication Type] | **994794** |
| **#4** | **#1 AND #2 Not #3** | **443** |

**Embase**

| No. | Query | Results |
| --- | --- | --- |
| #65 | #62 AND #64 | **2775** |
| #64 | trials:ti,kw,ab OR 'clinical trails':ti,kw,ab OR 'controlled trials':ti,kw,ab OR 'randomi?ed controlled trials':ti,kw,ab | **876886** |
| #63 | 'randomi?ed controlled trials':ti,kw,ab | **129483** |
| #62 | #60 NOT #61 | **17378** |
| #61 | review:ti OR 'case report':ti OR letter:ti OR editorial:ti OR comment:ti | **1148050** |
| #60 | #58 NOT #59 | **18297** |
| #59 | 'lung cancer':ti OR 'breast cancer':ti OR 'cervcal cancer':ti OR 'interview':ti OR 'meeting report':ti OR 'virus':ti OR 'test':ti OR 'detection':ti OR 'systematic review':ti OR 'meta analysis':ti | **1674978** |
| #58 | #56 NOT #57 | **18857** |
| #57 | 'dog':ti OR 'pig':ti OR 'rat':ti OR 'mice':ti OR 'mouse':ti OR 'animal':ti | **1410239** |
| #56 | #54 NOT #55 | **18896** |
| #55 | review:it OR 'case report':it OR letter:it OR editorial:it OR comment:it | **4737648** |
| #54 | #3 AND #53 | **25500** |
| #53 | #6 OR #9 OR #12 OR #15 OR #18 OR #21 OR #24 OR #27 OR #30 OR #33 OR #36 OR #39 OR #43 OR #46 OR #49 OR #52 | **184828** |
| #52 | #50 OR #51 | **1261** |
| #51 | 'amg 678':ti,ab,kw OR 'amg678':ti,ab,kw OR 'imlygic':ti,ab,kw OR 'oncovex':ti,ab,kw OR 'oncovex gm csf':ti,ab,kw OR 't vec':ti,ab,kw OR 'talminogene laherparepvec':ti,ab,kw | **444** |
| #50 | 'talimogene laherparepvec'/exp | **1169** |
| #49 | #47 OR #48 | **1717** |
| #48 | 'cik cell':ti,ab,kw OR 'cik cells':ti,ab,kw OR 'cytokine induced killer cells':ti,ab,kw OR 'cytokine-induced killer cells':ti,ab,kw | **1446** |
| #47 | 'cytokine induced killer cell'/exp | **1374** |
| #46 | #44 OR #45 | **1863** |
| #45 | '[3, 4 difluoro 2 (2 fluoro 4 iodoanilino) phenyl] [3 hydroxy 3 (2 piperidinyl) 1 azetidinyl] methanone':ti,ab,kw OR '[3, 4 difluoro 2 (2 fluoro 4 iodoanilino) phenyl] [3 hydroxy 3 (piperidin 2 yl) azetidin 1 yl] methanone':ti,ab,kw OR '[3, 4 difluoro 2 [ (2 fluoro 4 iodophenyl) amino] phenyl] [3 hydroxy 3 (2 piperidinyl) 1 azetidinyl] methanone':ti,ab,kw OR 'cobimetinib butyrate':ti,ab,kw OR 'cobimetinib fumarate':ti,ab,kw OR 'cobimetinib hemifumarate':ti,ab,kw OR 'cotellic':ti,ab,kw OR 'gdc 0973':ti,ab,kw OR 'gdc0973':ti,ab,kw OR 'rg 7420':ti,ab,kw OR 'rg7420':ti,ab,kw OR 'xl 518':ti,ab,kw OR 'xl518':ti,ab,kw | **97** |
| #44 | 'cobimetinib'/exp | **1824** |
| #43 | #40 OR #41 OR #42 | **35765** |
| #42 | 'checkpoint inhibitors, immune':ti,ab,kw OR 'immune checkpoint inhibitor':ti,ab,kw OR 'checkpoint inhibitor, immune':ti,ab,kw OR 'immune checkpoint blockers':ti,ab,kw OR 'checkpoint blockers, immune':ti,ab,kw OR 'immune checkpoint blockade':ti,ab,kw OR 'checkpoint blockade, immune':ti,ab,kw OR 'immune checkpoint inhibition':ti,ab,kw OR 'checkpoint inhibition, immune':ti,ab,kw OR 'pd-l1 inhibitors':ti,ab,kw OR 'pd l1 inhibitors':ti,ab,kw OR 'pd-l1 inhibitor':ti,ab,kw OR 'pd l1 inhibitor':ti,ab,kw OR 'programmed death-ligand 1 inhibitors':ti,ab,kw OR 'programmed death ligand 1 inhibitors':ti,ab,kw OR 'ctla-4 inhibitors':ti,ab,kw OR 'ctla 4 inhibitors':ti,ab,kw OR 'ctla-4 inhibitor':ti,ab,kw OR 'ctla 4 inhibitor':ti,ab,kw OR 'cytotoxic t-lymphocyte-associated protein 4 inhibitors':ti,ab,kw OR 'cytotoxic t lymphocyte associated protein 4 inhibitors':ti,ab,kw OR 'cytotoxic t-lymphocyte-associated protein 4 inhibitor':ti,ab,kw OR 'cytotoxic t lymphocyte associated protein 4 inhibitor':ti,ab,kw OR 'pd-1 inhibitors':ti,ab,kw OR 'pd 1 inhibitors':ti,ab,kw OR 'pd-1 inhibitor':ti,ab,kw OR 'inhibitor, pd-1':ti,ab,kw OR 'pd 1 inhibitor':ti,ab,kw OR 'programmed cell death protein 1 inhibitor':ti,ab,kw OR 'programmed cell death protein 1 inhibitors':ti,ab,kw OR 'pd-1-pd-l1 blockade':ti,ab,kw OR 'blockade, pd-1-pd-l1':ti,ab,kw OR 'pd 1 pd l1 blockade':ti,ab,kw | **20930** |
| #41 | 'immune checkpoint blocker':ti,ab,kw OR 'immune checkpoint inhibitors':ti,ab,kw | **18076** |
| #40 | 'immune checkpoint inhibitor'/exp | **8692** |
| #39 | #37 OR #38 | **3918** |
| #38 | 'bavencio':ti,ab,kw OR 'msb 0010682':ti,ab,kw OR 'msb 0010718c':ti,ab,kw OR 'msb 10682':ti,ab,kw OR 'msb 10718c':ti,ab,kw OR 'msb0010682':ti,ab,kw OR 'msb0010718c':ti,ab,kw OR 'msb10682':ti,ab,kw OR 'msb10718c':ti,ab,kw OR 'pf 06834635':ti,ab,kw OR 'pf 6834635':ti,ab,kw OR 'pf06834635':ti,ab,kw OR 'pf6834635':ti,ab,kw | **90** |
| #37 | 'avelumab'/exp | **3914** |
| #36 | #34 OR #35 | **771** |
| #35 | 'cemiplimab rwlc':ti,ab,kw OR 'cemiplimab-rwlc':ti,ab,kw OR 'libtayo; regn 2810':ti,ab,kw OR 'regn2810':ti,ab,kw OR 'sar 439684':ti,ab,kw OR 'sar439684':ti,ab,kw | **79** |
| #34 | 'cemiplimab'/exp | **760** |
| #33 | #31 OR #32 | **25850** |
| #32 | 'bms 936558':ti,ab,kw OR 'bms936558':ti,ab,kw OR 'cmab 819':ti,ab,kw OR 'cmab819':ti,ab,kw OR 'mdx 1106':ti,ab,kw OR 'mdx1106':ti,ab,kw OR 'ono 4538':ti,ab,kw OR 'ono4538':ti,ab,kw OR 'opdivo':ti,ab,kw | **333** |
| #31 | 'nivolumab'/exp | **25819** |
| #30 | #28 OR #29 | **23897** |
| #29 | 'keytruda':ti,ab,kw OR 'lambrolizumab':ti,ab,kw OR 'mk 3475':ti,ab,kw OR 'mk3475':ti,ab,kw OR 'sch 900475':ti,ab,kw OR 'sch900475':ti,ab,kw | **542** |
| #28 | 'pembrolizumab'/exp | **23845** |
| #27 | #25 OR #26 | **18299** |
| #26 | 'bms 734016':ti,ab,kw OR 'bms734016':ti,ab,kw OR 'mdx 010':ti,ab,kw OR 'mdx 101':ti,ab,kw OR 'mdx010':ti,ab,kw OR 'mdx101':ti,ab,kw OR 'strentarga':ti,ab,kw OR 'yervoy':ti,ab,kw | **196** |
| #25 | 'ipilimumab'/exp | **18285** |
| #24 | #22 OR #23 | **1378** |
| #23 | '5 (4 bromo 2 fluoroanilino) 4 fluoro n (2 hydroxyethoxy) 1 methyl 1h benzimidazole 6 carboxamide':ti,ab,kw OR '5 [ (4 bromo 2 fluorophenyl) amino] 4 fluoro n (2 hydroxyethoxy) 1 methyl 1h benzimidazole 6 carboxamide':ti,ab,kw OR 'arry 162':ti,ab,kw OR 'arry 438162':ti,ab,kw OR 'arry162':ti,ab,kw OR 'arry438162':ti,ab,kw OR 'balimek':ti,ab,kw OR 'mek 162':ti,ab,kw OR 'mek162':ti,ab,kw OR 'mektovi':ti,ab,kw | **191** |
| #22 | 'binimetinib'/exp | **1358** |
| #21 | #19 OR #20 | **917** |
| #20 | 'braftovi':ti,ab,kw OR 'lgx 818':ti,ab,kw OR 'lgx818':ti,ab,kw OR 'methyl n [1 [ [4 [3 [5 chloro 2 fluoro 3 (methanesulfonamido) phenyl] 1 (propan 2 yl) 1h pyrazol 4 yl] pyrimidin 2 yl] amino] propan 2 yl] carbamate':ti,ab,kw OR 'n [2 [ [4 [3 [5 chloro 2 fluoro 3 (methanesulfonamido) phenyl] 1 isopropyl 1h pyrazol 4 yl] 2 pyrimidinyl] amino] 1 methylethyl] carbamic acid methyl ester':ti,ab,kw OR 'n [2 [ [4 [3 [5 chloro 2 fluoro 3 [ (methylsulfonyl) amino] phenyl] 1 (1 methylethyl) 1h pyrazol 4 yl] 2 pyrimidinyl] amino] 1 methylethyl] carbamic acid methyl ester':ti,ab,kw OR 'nvp lgx 818':ti,ab,kw OR 'nvp lgx 818 nxa':ti,ab,kw OR 'nvp lgx818':ti,ab,kw OR 'nvp lgx818 nxa':ti,ab,kw | **65** |
| #19 | 'encorafenib'/exp | **902** |
| #18 | #16 OR #17 | **6675** |
| #17 | 'gsk 1120212':ti,ab,kw OR 'gsk 1120212b':ti,ab,kw OR 'gsk1120212':ti,ab,kw OR 'gsk1120212b':ti,ab,kw OR 'jtp 74057':ti,ab,kw OR 'jtp74057':ti,ab,kw OR 'mekinist':ti,ab,kw OR 'n [3 [3 cyclopropyl 5 (2 fluoro 4 iodoanilino) 6, 8 dimethyl 2, 4, 7 trioxopyrido [4, 3 d] pyrimidin 1 yl] phenyl] acetamide':ti,ab,kw OR 'n [3 [3 cyclopropyl 5 [ (2 fluoro 4 iodophenyl) amino] 3, 4, 6, 7 tetrahydro 6, 8 dimethyl 2, 4, 7 trioxopyrido [4, 3 d] pyrimidin 1 (2h) yl] phenyl] acetamide':ti,ab,kw OR 'n [3 [3 cyclopropyl 5 [ (2 fluoro 4 iodophenyl) amino] 6, 8 dimethyl 2, 4, 7 trioxo 3, 4, 6, 7 tetrahydropyrido [4, 3 d] pyrimidin 1 (2h) yl] phenyl] acetamide':ti,ab,kw OR 'snr 1611':ti,ab,kw OR 'snr1611':ti,ab,kw OR 'tmt 212':ti,ab,kw OR 'tmt212':ti,ab,kw OR 'trametinib dimethyl sulfoxide':ti,ab,kw | **217** |
| #16 | 'trametinib'/exp | **6647** |
| #15 | #13 OR #14 | **5438** |
| #14 | 'dabrafenib mesylate':ti,ab,kw OR 'drb 436':ti,ab,kw OR 'drb436':ti,ab,kw OR 'gsk 2118436':ti,ab,kw OR 'gsk 2118436a':ti,ab,kw OR 'gsk 2118436b':ti,ab,kw OR 'gsk2118436':ti,ab,kw OR 'gsk2118436a':ti,ab,kw OR 'gsk2118436b':ti,ab,kw OR 'n [3 [5 (2 amino 4 pyrimidinyl) 2 (1, 1 dimethylethyl) 1, 3 thiazol 4 yl] 2 fluorophenyl] 2, 6 difluorobenzenesulfonamide':ti,ab,kw OR 'n [3 [5 (2 amino 4 pyrimidinyl) 2 (1, 1 dimethylethyl) 4 thiazolyl] 2 fluorophenyl] 2, 6 difluorobenzenesulfonamide':ti,ab,kw OR 'n [3 [5 (2 amino 4 pyrimidinyl) 2 tert butyl 4 thiazolyl] 2 fluorophenyl] 2, 6 difluorobenzenesulfonamide':ti,ab,kw OR 'n [3 [5 (2 aminopyrimidin 4 yl) 2 tert butyl 1, 3 thiazol 4 yl] 2 fluorophenyl] 2, 6 difluorobenzenesulfonamide':ti,ab,kw OR 'tafinlar':ti,ab,kw | **134** |
| #13 | 'dabrafenib'/exp | **5411** |
| #12 | #10 OR #11 | **8782** |
| #11 | 'n [3 [5 (4 chlorophenyl) 1h pyrrolo [2, 3 b] pyridine 3 carbonyl] 2, 4 difluorophenyl] propanesulfonamide':ti,ab,kw OR 'n [3 [ [5 (4 chlorophenyl) 1h pyrrolo [2, 3 b] pyridin 3 yl] carbonyl] 2, 4 difluorophenyl] 1 propanesulfonamide':ti,ab,kw OR 'plx 4032':ti,ab,kw OR 'plx4032':ti,ab,kw OR 'r 7204':ti,ab,kw OR 'r7204':ti,ab,kw OR 'rg 7204':ti,ab,kw OR 'rg7204':ti,ab,kw OR 'ro 5185426':ti,ab,kw OR 'ro5185426':ti,ab,kw OR 'zelboraf':ti,ab,kw | **589** |
| #10 | 'vemurafenib'/exp | **8734** |
| #9 | #7 OR #8 | **49419** |
| #8 | 'molecular target therapy':ti,ab,kw OR 'molecular targeted therapy':ti,ab,kw OR 'targeted cancer therapy':ti,ab,kw OR 'targeted molecular therapy':ti,ab,kw OR 'targeted therapy (cancer)':ti,ab,kw | **6234** |
| #7 | 'molecularly targeted therapy'/exp | **46298** |
| #6 | #4 OR #5 | **62918** |
| #5 | 'chemotherapy, adjuvant':ti,ab,kw | **775** |
| #4 | 'adjuvant chemotherapy'/exp | **62430** |
| #3 | #1 OR #2 | **185253** |
| #2 | 'fortner melanoma':ti,ab,kw OR 'malignant melanoma':ti,ab,kw OR 'malignant melanomatosis':ti,ab,kw OR 'melanocarcinoma':ti,ab,kw OR 'melanoma (e)':ti,ab,kw OR 'melanomalignoma':ti,ab,kw OR 'naevi and melanomas':ti,ab,kw OR 'naevocarcinoma':ti,ab,kw OR 'nevi and melanomas':ti,ab,kw OR 'nevocarcinoma':ti,ab,kw OR 'nodular melanoma':ti,ab,kw OR 'pigmentary cancer':ti,ab,kw | **36364** |
| #1 | 'melanoma'/exp | **181098** |

| **Cochrane** | | |
| --- | --- | --- |
| **Step** | **Search manager** | **Items found** |
| **#1** | MeSH descriptor: [Melanoma] explode all trees | **1901** |
| **#2** | (“Melanomas” OR “Malignant Melanoma” OR “Malignant Melanomas” OR “Melanoma, Malignant” OR “Melanomas, Malignant”)ti,ab,kw | **30** |
| **#3** | #1 OR #2 | **1929** |
| **#4** | **MeSH descriptor: [Chemotherapy, Adjuvant] explode all trees** | **4013** |
| **#5** | (“Drug Therapy, Adjuvant” OR “Adjuvant Chemotherapy” OR “Adjuvant Drug Therapy”)ti,ab,kw | **22** |
| **#6** | #4 OR #5 | **4031** |
| **#7** | MeSH descriptor: [Molecular Targeted Therapy] explode all trees | **150** |
| **#8** | (“Molecular Targeted Therapies” OR “Targeted Therapy, Molecular” OR “Therapy, Molecular Targeted” OR “Targeted Molecular Therapy” OR “Molecular Therapy, Targeted” OR “Targeted Molecular Therapies” OR “Therapy, Targeted Molecular”)ti,ab,kw | **2** |
| **#9** | #7 OR #8 | **151** |
| **#10** | MeSH descriptor: [Vemurafenib] explode all trees | **47** |
| **#11** | (“PLX4032” OR “PLX 4032” OR “RG7204” OR “RG-7204” OR “RG 7204” OR “Zelboraf” OR “R05185426”)ti,ab,kw | **0** |
| **#12** | #10 OR #11 | **47** |
| **#13** | MeSH descriptor: [Ipilimumab] explode all trees | **219** |
| **#14** | (“Anti-CTLA-4 MAb Ipilimumab” OR “Anti CTLA 4 MAb Ipilimumab” OR “Ipilimumab, Anti-CTLA-4 MAb” OR “Yervoy” OR “MDX 010” OR “MDX010” OR “MDX-010” OR “MDX-CTLA-4” OR “MDX CTLA 4”)ti,ab,kw | **132** |
| **#15** | #15 #13 OR #14 | **220** |
| **#16** | MeSH descriptor: [Nivolumab] explode all trees | **516** |
| **#17** | (“Opdivo” OR “ONO-4538” OR “ONO 4538” OR “ONO4538” OR “MDX-1106” OR “MDX 1106” OR “MDX1106” OR “BMS-936558” OR “BMS 936558” OR “BMS936558”)ti,ab,kw | **2** |
| **#18** | #16 OR #17 | **518** |
| **#19** | MeSH descriptor: [Immune Checkpoint Inhibitors] explode all trees | **32** |
| **#20** | (Checkpoint Inhibitors, Immune):ti,ab,kw OR (Immune Checkpoint Inhibitor):ti,ab,kw OR (Checkpoint Inhibitor, Immune):ti,ab,kw OR (Immune Checkpoint Blockers):ti,ab,kw OR (Checkpoint Blockers, Immune):ti,ab,kw (Word variations have been searched) | **1245** |
| **#21** | (Immune Checkpoint Blockade):ti,ab,kw OR (Checkpoint Blockade, Immune):ti,ab,kw OR (Immune Checkpoint Inhibition):ti,ab,kw OR (Checkpoint Inhibition, Immune):ti,ab,kw OR (PD-L1 Inhibitors):ti,ab,kw (Word variations have been searched) | **1416** |
| **#22** | (PD L1 Inhibitors):ti,ab,kw OR (PD-L1 Inhibitor):ti,ab,kw OR (PD L1 Inhibitor):ti,ab,kw OR (Programmed Death-Ligand 1 Inhibitors):ti,ab,kw OR (Programmed Death Ligand 1 Inhibitors):ti,ab,kw (Word variations have been searched) | **1077** |
| **#23** | (CTLA-4 Inhibitors):ti,ab,kw OR (CTLA 4 Inhibitors):ti,ab,kw OR (CTLA-4 Inhibitor):ti,ab,kw OR (CTLA 4 Inhibitor):ti,ab,kw OR (Cytotoxic T-Lymphocyte-Associated Protein 4 Inhibitors):ti,ab,kw (Word variations have been searched) | **257** |
| **#24** | (Cytotoxic T Lymphocyte Associated Protein 4 Inhibitors):ti,ab,kw OR (Cytotoxic T-Lymphocyte-Associated Protein 4 Inhibitor):ti,ab,kw OR (Cytotoxic T Lymphocyte Associated Protein 4 Inhibitor):ti,ab,kw OR (CTLA 4 Inhibitor):ti,ab,kw OR (PD-1 Inhibitors):ti,ab,kw (Word variations have been searched) | **1181** |
| **#25** | (PD 1 Inhibitors):ti,ab,kw OR (PD-1 Inhibitor):ti,ab,kw OR (Inhibitor, PD-1):ti,ab,kw OR (PD 1 Inhibitor):ti,ab,kw OR (Programmed Cell Death Protein 1 Inhibitor):ti,ab,kw (Word variations have been searched) | **5391** |
| **#26** | (Programmed Cell Death Protein 1 Inhibitors):ti,ab,kw (Word variations have been searched) | **235** |
| **#27** | #19 OR #20 OR #21 OR #22 OR #23 OR #24 OR #25 OR #26 | **6269** |
| **#28** | MeSH descriptor: [Cytokine-Induced Killer Cells] explode all trees | **276** |
| **#29** | (“Cytokine Induced Killer Cells” OR “Cytokine-Induced Killer Cell” OR “Killer Cell, Cytokine-Induced” OR “Killer Cells, Cytokine-Induced” OR “CIK Cells” OR “CIK Cell” OR “Lymphocyte-Activated Killer Cells” OR “Cell, Lymphocyte-Activated Killer” OR “Cells, Lymphocyte-Activated Killer” OR “Killer Cell, Lymphocyte-Activated” OR “Killer Cells, Lymphocyte-Activated” OR “Lymphocyte Activated Killer Cells” OR “Lymphocyte-Activated Killer Cell” OR “Lymphocyte Activated Killer Cell”)ti,ab,kw | **0** |
| **#30** | #28 OR #29 | **276** |
| **#31** | #6 OR #9 OR #12 OR #15 OR #18 OR #27 OR #30 | **11197** |
| **#32** | #3 AND #31 | **357** |
